# Immunotherapy for triple-negative breast cancer: current trends and future prospects

**DOI:** 10.1186/s43046-025-00295-x

**Published:** 2025-06-17

**Authors:** Amina Essalihi, Oumaima Bouchra, Khadija Khadiri, Zineb Khadrouf, Mehdi Karkouri

**Affiliations:** 1 Laboratory of Cellular and Molecular Pathology, Faculty of Medicine and Pharmacy, Hassan II University of Casablanca, Casablanca, Morocco; 2 Laboratory of Immunology and Biodiversity, Faculty of Sciences, Aïn Chock, Hassan II University of Casablanca, Casablanca, Morocco; 3 Pathology Department, University Hospital Ibn Rochd, Casablanca, Casablanca, Morocco

**Keywords:** Immunotherapy, Triple-negative breast cancer, Immune checkpoint inhibitors, Clinical Trials, PDL-1, Tumor-infiltrating lymphocytes

## Abstract

Triple-negative breast cancer (TNBC) accounts for 10–20% of all breast cancers. These tumors are heterogeneous, highly aggressive, and associated with a poor prognosis and a high risk of recurrence. In both hematologic and solid malignancies, immune checkpoint inhibitors (ICIs) have demonstrated the ability to enhance long-term survival and sustain robust anti-tumor responses. Immunotherapy has also been introduced as a treatment option for TNBC, a subtype characterized by a high presence of intra-tumoral tumor-infiltrating lymphocytes (TILs) and stromal immune cells. This heightened immune activity within TNBC serves as a prognostic marker, indicating a potential for better responses to immunotherapy due to increased tumor immune infiltration. This review provides an overview of the current landscape of immunotherapy in TNBC, exploring its rationale and application across different disease stages.

**Trial registration** NCT02555657.

## Introduction

Breast cancers are classified into different subcategories to guide their management. Triple-negative breast cancer (TNBC) is diagnosed when no known marker responsive to targeted therapy is identified [1]. It is characterized by the absence of human epidermal growth factor receptor 2 (HER2) overexpression/amplification, as well as the absence of estrogen receptors (ER) and progesterone receptors (PR) [2]. These cancers, which represent approximately 10–20% of all breast cancers, are highly aggressive, and the current treatment options remain insufficiently effective [3, 4].

The management of TNBC relies on locoregional treatments such as surgery and radiotherapy. Additionally, neoadjuvant chemotherapy (NACT) is frequently used for larger tumors to facilitate breast-conserving surgery [5].

However, despite chemotherapy (CHT) intervention, TNBC remains associated with early recurrence and high morbidity. Compared to HER2-positive and hormone receptor-positive breast tumors, TNBC patients continue to have poorer prognoses [6].

Immunotherapy has emerged as a potential new therapeutic option for TNBC patients. Programmed death-ligand 1 (PD-L1) is a key immune checkpoint protein that helps regulate immune responses. Checkpoint inhibitors (ICIs), a type of immunotherapy, work by blocking PD-L1, thereby preventing cancer cells from evading immune system attacks [5].

Checkpoint blockade in melanoma has been highly successful, prompting the investigation of these agents in other malignancies, including TNBC [7].

Several biological factors contribute to TNBC’s increased responsiveness to immunotherapy. High levels of tumor-infiltrating lymphocytes (TILs) and PD-L1 expression [8], as well as a greater tumor mutational burden (TMB) [9], play key roles.

However, identifying reliable predictive biomarkers of response is becoming increasingly important to optimize precision immunotherapy [10]. This is particularly crucial in early-stage TNBC, where the goal is not only achieving a cure but also minimizing the risk of long-term side effects from immunotherapy [10].

**Fig. 1:**
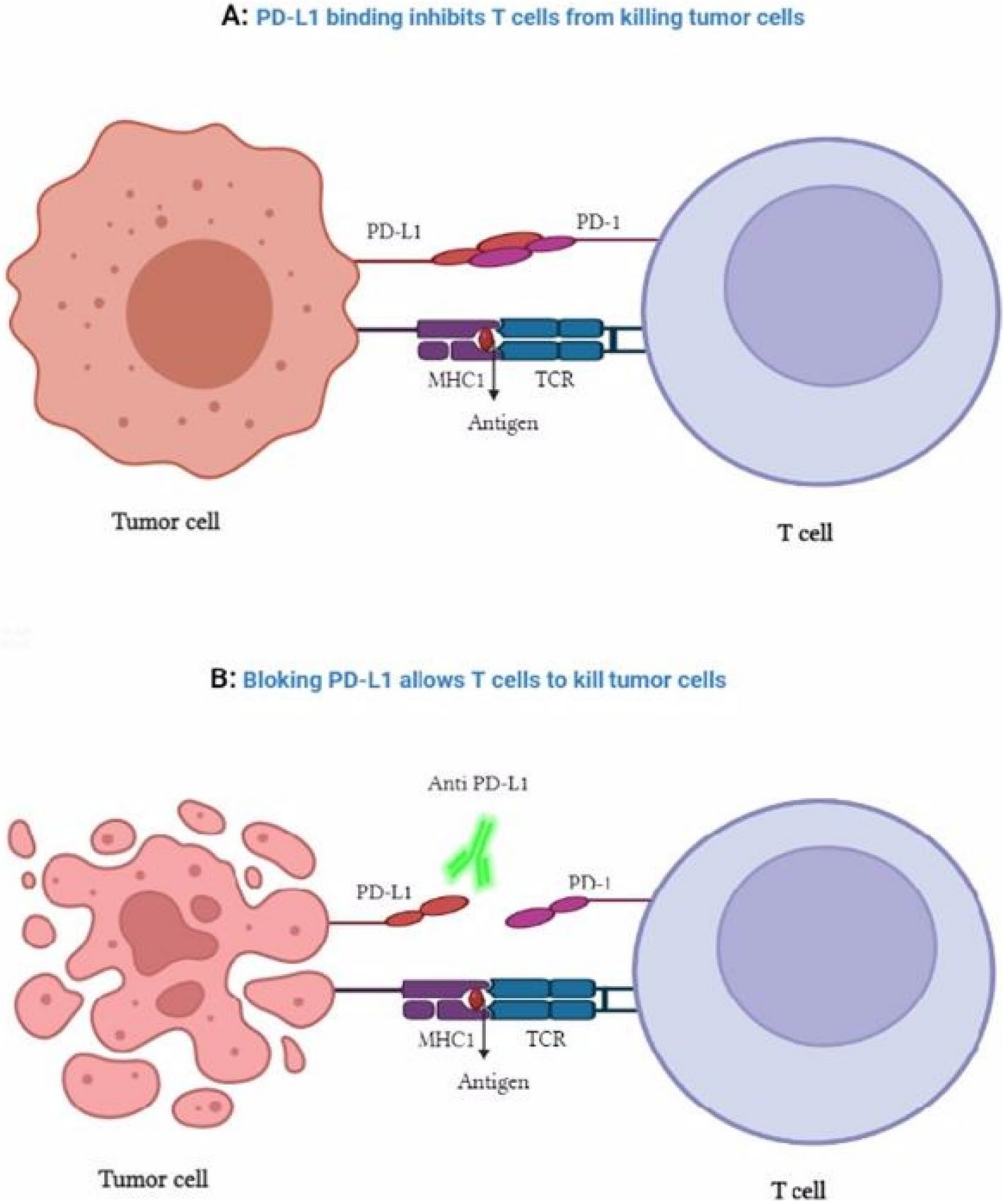
Mechanism of anti-PD-L1 antibodies

This review summarizes the current clinical experience with ICIs in both advanced and early-stage TNBC, highlighting the challenges of biomarker-based patient selection (Fig. 1).

### Immune checkpoint inhibitors as monotherapy

ICI response rates were higher in TNBC than in HER2-positive and luminal subtypes, according to early studies [6]. These findings were particularly significant in tumors expressing PD-L1 and with high levels of TILs (Table 1) [11].

**Table 1:** Immune checkpoint blockade in metastatic TNBC trials, with or without chemotherapy

Trial	Treatment	PD-L1 expression	*N*	ORR (%)	Median PFS (months)	Median OS (months)
KEYNOTE-012	Pembrolizumab	≥ 1% tumor cell (TC) or stroma	32	18.5	1.9	11.2
JAVELIN	Avelumab	~ 50% PD-L1 positive	58	5.2	5.9	9.2
JAVELIN	Atezolizumab	~ 50% PD-L1 positive	115	10	1.4	8.9
		PD-L1 IC ≥ 1%	91	12	1.4	10.1
		PD-L1 IC < 1%	21	0	1.4	6.0
KEYNOTE-086 A	Pembrolizumab	~ 40% PD-L1 CPS ≥ 1	170	5.3	2.0	9.0
		~ 60% PD-L1 CPS < 1	64	4.7	1.9	9.7
KEYNOTE-086B	Pembrolizumab	CPS ≥ 1	84	21.4	2.1	18.0
KEYNOTE-119	Pembrolizumab vs Chemotherapy	CPS ≥ 1 (39% positive)	622	9.6 vs 10.6	2.1 vs 3.3	9.9 vs 10.8
		CPS ≥ 1	405	12.3 vs 9.4	2.1 vs 3.1	10.7 vs 10.2
		CPS ≥ 10	194	17.7 vs 9.2	2.1 vs 3.4	12.7 vs 11.6
		CPS ≥ 20	109	26.3 vs 11.5	3.4 vs 2.4	14.9 vs 12.5
SAFIR02-BREAST IMMUNO	Maintenance durvalumab	~ 45% PD-L1 positive	82	NA	HR 0.87 vs HR 1.00	21 vs 14

*Abbreviations*: *CPS* combined positive score, *DFS*, disease-free survival, *IC* immune cells; NA, not available, *ORR* overall response rate, *OS* overall survival, *PD-L1* programmed death-ligand 1, *PFS* progression-free survival, *TC* tumor cells, *TNBC* triple-negative breast cancer

The KEYNOTE-012 clinical study reported an overall response rate (ORR) of 18.5%. This trial included 32 patients with advanced TNBC and positive PD-L1 expression who received pembrolizumab monotherapy. The treatment demonstrated durable anticancer activity and manageable toxicity in metastatic TNBC (mTNBC) patients [12, 13]. Notwithstanding this encouraging beginning, further research has shown a number of drawbacks, such as variations in patient selection, variations in PD-L1 testing techniques, and a dearth of reliable biomarkers to forecast ICI response.

For example, multi-cohort trials for mTNBC investigated PD-L1 inhibitors like atezolizumab and avelumab. In the phase Ib JAVELIN study, avelumab showed an ORR of only 5.2% in 58 TNBC patients, with a median of three prior treatments for advanced disease [14, 15]. These findings show that single-agent ICIs are not very effective in populations that have had a lot of pretreatment, and that combination strategies are needed to boost response rates [16].

In the phase II KEYNOTE-086 trial, 170 mTNBC patients who had previously received treatment were studied. Cohort A reported ORRs of 5.7% in PD-L1 + and 4.7% in PD-L1 − patients [17]. In contrast, Cohort B, which included 84 treatment-naive PD-L1 + patients, showed an ORR of 21.4%, suggesting that ICIs may be more effective as a first-line therapy for mTNBC [18]. This highlights the importance of patient stratification and timing in the administration of ICIs.

Despite the high risk of recurrence in TNBC, it remains highly chemosensitive [5]. The KEYNOTE-119 trial compared pembrolizumab monotherapy with chemotherapy in 622 patients with advanced TNBC. This phase III trial (NCT02555657) revealed no significant differences in overall survival (OS) or progression-free survival (PFS) between the two treatments. However, pembrolizumab demonstrated improved ORRs in patients with higher PD-L1 expression, particularly in the combined positive score (CPS) ≥ 10 group (ORR 17.7%) compared to the CPS ≥ 1 group (ORR 12.3%) [19, 20]. This indicates a correlation between higher PD-L1 expression and improved response to pembrolizumab.

When compared to chemotherapy, pembrolizumab also showed a good safety profile in patients with increased PD-L1 expression, with fewer grade 3 to 5 adverse events and better quality of life outcomes [21]. These results highlight its potential as a safer alternative to chemotherapy, particularly in patients with high PD-L1 expression.

In the phase II SAFIR02-IMMUNO trial, 199 HER2-negative metastatic breast cancer patients received either maintenance chemotherapy or durvalumab after 6–8 cycles of initial treatment. Durvalumab did not significantly improve PFS in the overall study population. However, an exploratory analysis of 82 TNBC patients revealed a median OS benefit (21.2 months vs. 14.0 months; HR 0.54; *p* = 0.0377) and a trend toward improved PFS (HR 0.87; 95% CI 0.54–1.42) [22, 23]. These findings suggest that anti-PD-L1 immunotherapy may offer benefits in selected TNBC patients, irrespective of PD-L1 receptor status.

While ICIs have shown promise, their limited efficacy in unselected patient populations highlights several challenges. One major limitation is the heterogeneity of TNBC, which comprises distinct molecular subtypes with variable immunogenicity. Future studies should focus on identifying robust predictive biomarkers beyond PD-L1, such as TIL density, TMB, or gene expression profiles associated with immune activation.

Moreover, combination strategies, such as ICIs with chemotherapy, anti-angiogenic agents, or other immunomodulators, may enhance efficacy. For example, the IMpassion130 trial demonstrated a significant improvement in PFS and OS when atezolizumab was combined with nab-paclitaxel in PD-L1 + mTNBC patients [24].

Innovative approaches, such as targeting other immune checkpoints (e.g., LAG-3, TIM-3) or utilizing personalized neoantigen vaccines, hold great potential. Additionally, the integration of multi-omics data could provide a deeper understanding of TNBC immunobiology and guide the development of more effective therapeutic strategies [25, 26].

### Metastatic chemotherapy ombination regimens

While some patients who received ICIs as monotherapy experienced significant benefits, these findings highlight the necessity of combination regimens to improve therapeutic outcomes (Table 2). In a phase 1b study, atezolizumab and nab-paclitaxel were administered to 33 TNBC patients, yielding an ORR of 39.4% [27]. This encouraging result paved the way for the phase III IMpassion130 trial (NCT02425891) [28]. IMpassion130 was a large-scale, randomized, double-blind, placebo-controlled study that enrolled 902 patients with untreated mTNBC. The trial demonstrated a median PFS improvement of 7.2 months in the atezolizumab-nab-paclitaxel arm compared to 5.5 months in the placebo-nab-paclitaxel arm in the PD-L1-positive subgroup. Furthermore, a 7-month OS benefit was observed in this subgroup [29]. A statistically significant OS benefit was not found in the overall cohort analysis because of the combination’s limited effectiveness in PD-L1-negative patients.

**Table 2:** Immunotherapy randomized clinical trials for advanced triple-negative breast cancer

Trial	Treatment	PD-L1 expression	*N*	ORR (%)	Median PFS (months)	Median OS (months)
IMpassion130	Atezolizumab + nab-paclitaxel	~ 50% PD-L1 positive	902	56.0 vs 45.9	7.2 vs 5.5	21.0 vs 18.7
	Placebo + nab-paclitaxel	PD-L1 IC ≥ 1%	369	58.9 vs 42.6	7.5 vs 5.0	25.0 vs 18.0
IMpassion131	Atezolizumab + paclitaxel	~ 50% PD-L1 positive	651	54 vs 47	5.7 vs 5.6	19.2 vs 22.8
	Placebo + paclitaxel	PD-L1 IC ≥ 1%	292	63 vs 55	6.0 vs 5.7	22.1 vs 28.3
KEYNOTE-355	Chemotherapy (carboplatin + gemcitabine, or nab-/paclitaxel) + Pembrolizumab	~ 50% PD-L1 positive	847	40.8 vs 37.0	7.5 vs 5.6	17.2 vs 15.5
	Chemotherapy (carboplatin + gemcitabine, or nab-/paclitaxel) + Placebo	CPS ≥ 1	636	44.9 vs 38.9	7.6 vs 5.6	17.6 vs 16.0
		CPS ≥ 10	323	52.7 vs 40.8	9.7 vs 5.6	23.0 vs 16.1

*Abbreviations*: *CPS* combined positive score, *DFS* disease-free survival, *IC* immune cells, *NA* not available, *ORR* overall response rate, *OS* overall survival, *PD-L1* programmed death-ligand 1, *PFS* progression-free survival, *TC* tumor cells, *TNBC* triple-negative breast cancer

The FDA gave atezolizumab and nab-paclitaxel accelerated approval for PD-L1-positive mTNBC based on the IMpassion130 results. However, subsequent trials, such as IMpassion131, failed to replicate these findings, leading to the withdrawal of this approval. IMpassion131 compared atezolizumab with paclitaxel versus paclitaxel alone in 651 patients and found no significant improvement in PFS in the PD-L1-positive population (median 6.0 months vs. 5.7 months; HR 0.82, *p* = 0.20) [30]. Concerns were raised regarding the validity of PD-L1 as a biomarker and the possible impact of chemotherapy agents (paclitaxel vs. nap-paclitaxel) on immunotherapy outcomes due to the trial’s lack of OS or PFS improvement.

The KEYNOTE-355 trial, another important study, assessed pembrolizumab in combination with chemotherapy in 847 patients with TNBC that was inoperable, locally recurrent, or showed a significant improvement in PFS for patients in the CPS ≥ 10 subgroup receiving pembrolizumab-chemotherapy compared to chemotherapy alone [31]. These findings highlighted the importance of patient stratification and the potential for ICIs to provide durable benefits when combined with chemotherapy as a first-line therapy in selected patients.

Despite promising results, several limitations persist in the use of ICIs combined with chemotherapy for TNBC. One of the main challenges is the inconsistency in clinical outcomes, as seen in the contrasting findings of IMpassion130 and IMpassion131 [32]. This discrepancy may be attributed to variations in chemotherapy backbones or differences in patient populations. Additionally, PD-L1 expression remains an imperfect biomarker, as its predictive value is not always consistent across studies, emphasizing the need for alternative predictive markers such as TMB, TIL density, or immune gene signatures. Furthermore, ICIs tend to show reduced efficacy in heavily pretreated patients, underscoring the importance of early intervention.

Single-cell RNA sequencing and spatial transcriptomics could help refine patient stratification by identifying distinct immune landscapes within TNBC [33]. Additionally, targeting the immunosuppressive tumor microenvironment (TME) through the depletion of regulatory T cells or inhibition of myeloid-derived suppressor cells may optimize the therapeutic potential of ICIs [34].

For example, research suggests that the distance between APOE + macrophages and exhausted CD8 + T cells (Tex) may play a key role in ICI efficacy. A study showed that in TNBC responding to ICIs, the distance between these cells was greater compared to untreated patients [35]. This indicates that the distribution of immune cells within the TME could be a crucial factor determining the success of immunotherapy.

### Early-stage triple-negative breast cancer combination regimens

Early TNBC patients treated with pembrolizumab and neoadjuvant chemotherapy (NACT) have shown promising results.

In the I-SPY2 trial (NCT01042379), 69 patients with high-risk hormone receptor-positive (HR +) breast cancer and 29 TNBC patients were randomized to receive 4 cycles of pembrolizumab plus weekly NACT, while 181 patients were assigned to the control group receiving standard NACT [31]. The initial results demonstrated that the pathologic complete response (pCR) rate increased from 22% in the control arm to 60% in the pembrolizumab group among TNBC patients, suggesting a substantial benefit from adding pembrolizumab to NACT [36].

The TONIC trial (NCT02499367) examined different induction therapies before nivolumab was administered to 67 patients with mTNBC [32]. Patients were randomly assigned to receive nivolumab either by itself or in conjunction with low-dose cyclophosphamide, cisplatin, or doxorubicin, or short-term induction with irradiation (3 × 8 Gy). The highest objective response rate (ORR) was observed in the doxorubicin induction group (35%), indicating that doxorubicin may enhance susceptibility to PD-1 blockade in TNBC [37].

The GeparNuevo study (NCT02685059), a randomized, placebo-controlled, double-blind phase II trial, evaluated the addition of durvalumab to neoadjuvant chemotherapy in early-stage TNBC [33, 34]. In the serological phase involving 117 patients, the pCR rate was 44.2% in the durvalumab arm, but statistical significance was not reached compared to placebo (*p* = 0.287). However, patients with high TIL density exhibited significantly higher pCR rates (*p* < 0.01). The study also suggested that initiating durvalumab 2 weeks before chemotherapy might improve pCR rates, though these findings require further validation [38].

In the KEYNOTE-173 trial, the combination of pembrolizumab with neoadjuvant chemotherapy, with or without carboplatin, demonstrated significant antitumor activity in mTNBC [39]. The pCR rate was 60% (90% CI, 30–85), suggesting that PD-L1 expression, TIL density, and other immune biomarkers might be associated with treatment response [39].

The results of KEYNOTE-522, a randomized, double-blind phase III trial, demonstrated a significant clinical benefit with a pCR rate of 64.8% in the pembrolizumab group compared to 51.2% in the placebo group (*p* < 0.001). Notably, pembrolizumab showed efficacy even in patients with lower PD-L1 expression, contrasting with findings from IMpassion130, which demonstrated efficacy mainly in PD-L1-positive TNBC cases [40].

However, the NeoTRIPaPDL1 (NCT02620280) results, which included 280 patients with locally advanced or high-risk TNBC, did not show a statistically significant difference in pCR rates between the atezolizumab-treated group and the control group (43.5% vs. 40.8%, *p* = 0.66).

It is noteworthy that anthracycline administration occurred postoperatively in this study, raising questions about the impact of treatment sequencing and the timing of immunotherapy administration on efficacy [41].

While clinical trials generally indicate the significant potential of ICIs in combination with NACT, several limitations must be addressed. One major challenge lies in the heterogeneity of trial results, as demonstrated by the discrepancy between KEYNOTE-522, which showed a clear benefit of pembrolizumab, and NeoTRIPaPDL1, which did not observe an improvement in pCR with atezolizumab. These discrepancies emphasize the need for standardizing study methodologies and treatment protocols to optimize result comparability and clinical applicability [40, 42, 43].

Moreover, while PD-L1 expression is a commonly used biomarker, it remains an imperfect predictor of ICI response. Another major obstacle in evaluating ICIs is the lack of long-term follow-up, as many studies have yet to provide robust data on OS and PFS. These parameters are crucial for assessing the durability of immune responses and guiding future therapeutic strategies [44].

More research is necessary to fully understand the role of short-term induction therapies, as examined in the TONIC study. Specifically, it is important to ascertain whether these initial treatments have the ability to alter the TME and enhance ICI responses. For real-time analysis of TIL fluctuations, PD-L1 expression, and other immune markers during treatment, longitudinal biomarker studies are also essential. These analyses could provide crucial insights into tumor resistance mechanisms and enable personalized treatment adjustments based on each patient’s evolving profile [37].

Finally, while patient selection is currently heavily reliant on PD-L1 expression, alternative strategies should be considered to include PD-L1-negative patients, who might benefit from combination regimens targeting other aspects of the TME [40].

CTLA-4 inhibitors, including tremelimumab and ipilimumab, are also being investigated for their potential in the treatment of TNBC in addition to PD-1/PD-L1 inhibitors. In phase II clinical trials, the CTLA-4 inhibitor tremelimumab is being used in conjunction with chemotherapies like carboplatin, gemcitabine, and nab-paclitaxel to treat mTNBC. This treatment is also being tested with other immunotherapies like durvalumab and polyICLC. These studies aim to explore possible synergies between CTLA-4 inhibitors and other treatments to overcome resistance and improve clinical responses [45].

Ipilimumab, another CTLA-4 inhibitor, is being studied in various clinical trials, often in combination with PD-1 inhibitors like nivolumab or pembrolizumab. These trials are conducted for advanced breast cancers, including those negative for estrogen receptors (ER −), progesterone receptors (PR −), and HER2 [46, 47].

According to the current data, patients with TNBC may experience a higher percentage of full pathological responses when ICIs are added to NACT [48]. In another NeoTRIPaPDL1 trial (NCT02620280), 280 patients who were at high risk or locally advanced were recently assigned to receive either atezolizumab or neoadjuvant carboplatin and nab-paclitaxel. Atezolizumab and controls did not substantially alter the pCR rates (43.5% versus 40.8%, respectively, *p* = 0.66) [39]. The results compared to NeoTRIPaPDL1 might suggest that immunotherapy and anthracyclines complement each other because anthracyclines were only administered postoperatively in NeoTRIPaPDL1 (Table 3).

**Table 3:** Combination therapy trials in early-stage TNBC

Trial	Treatment arms	Subgroups	*N*	pCR (%) (95% CI)
NCT01042379a (I-SPY2)	T + pembrolizumab × 4 → AC × 4 → surgery vs T × 4 → AC × 4 → surgery	ITT	29 vs 85	60 vs 22
	T + pembrolizumab × 4 → pembrolizumab × 4 vs T × 4 → AC × 4	ITT	73 vs 295	27 vs 27
GeparNuevo NCT02685059	nab-T + durvalumab × 4 → EC + durvalumab × 4	ITT	174	53.4 vs 44.2
	Window: durvalumab/placebo 2 weeks before nab-paclitaxel	ITT	117	61.0 vs 41.4
KEYNOTE-173 NCT02622074	Nab-/paclitaxel ± carboplatin + pembrolizumab → AC + pembrolizumab → surgery	ITT	60	60
KEYNOTE-522 NCT03036488	Paclitaxel + carboplatin × 4 ± pembrolizumab × 4 → AC/EC ± pembrolizumab × 4 → surgery ± pembrolizumab × 9 cycles	ITT	1174	64.8 vs 51.2
NeoTRIPaPDL1 NCT02620280	Carboplatin + nab-paclitaxel ± atezolizumab × 8 → surgery → AC/EC/FEC × 4 cycles	ITT	280	43.5 vs 40.8

*Abbreviations*: *A* doxorubicin, *C* cyclophosphamide, *CT* control, *E* epirubicin, *F* 5-fluorouracil, *IDC* invasive ductal carcinoma, *IN* intervention, *ITT* intention to treat, *pCR* pathologic complete response, *PD-L1* programmed death-ligand 1, *T* paclitaxel

With the best response rates following doxorubicin induction in patients with mTNBC, the TONIC study further confirmed these findings. This trial proved that doxorubicin was preferable as an induction drug for increasing sensitivity to PD-1 inhibitors.

### Combining targeted therapies

To treat intrinsic resistance to PD-L1/PD-1 inhibitors in TNBC patients, a number of targeted combination therapies have been studied (Table 4). In patients with BRCA1 or BRCA2 mutations, poly (ADP-ribose) polymerase (PARP) inhibitors, which target deficiencies in homologous recombination repair (HRR), show great promise. These inhibitors are presently being tested in a number of contexts, such as mTNBC [49, 50], adjuvant, and neoadjuvant.

**Table 4:** Targeted therapy combination trials

Trial	Treatment	Biomarker	*N*	ORR (%)	Median PFS (months)	Median OS (months)
TOPACIO/KEYNOTE-162 NCT02657889	Niraparib + pembrolizumab	PD-L1 positive or negative, BRCAm positive or negative	55	21	2.3	-
		BRCAm positive	15	47	8.3	-
		BRCAm negative	27	11	2.1	-
MEDIOLA NCT02734004	Olaparib + durvalumab after 4 wk run-in	Germline BRCAm positive	17	58.8	4.9	20.5
Schmid AACR NCT03800836	Nab-/paclitaxel + ipatasertib + atezolizumab	PD-L1 positive or negative	26	73	-	-
COLET NCT02322814	Nab-paclitaxel vs paclitaxel + cobimetinib + atezolizumab	PD-L1 positive or negative	90	29.0 vs 34.4	7.0 vs 3.8	NR vs 11.0

*Abbreviations*: *BRCAm* BRCA mutation, *NR* not reached, *ORR* overall response rate, *OS* overall survival, *PFS* progression-free survival, *TNBC* triple-negative breast cancer

Comparable to single-agent PARP therapy, the MEDIOLA trial, which paired durvalumab with olaparib in patients with HER2-negative metastatic breast cancer who had BRCA mutations, demonstrated an ORR of 58.8% and a median PFS of 4.9 months. These findings raise questions about the additional benefit of combining these agents [51, 52].

AKT inhibitors are also promising in TNBC; a phase Ib study showed that, independent of biomarker status, the first 26 patients treated with ipatasertib, atezolizumab, and chemotherapy had an amazing ORR of 73%. AKT inhibition targets the PI3 K/AKT/mTOR pathway, which is frequently altered in TNBC, suggesting another potential avenue for effective combination therapies [53].

Additionally, MEK inhibitors combined with ICIs have generated significant interest. In 63 mTNBC patients who had not yet received treatment, the phase II COLET trial assessed the effects of cobimetinib, a MEK inhibitor, in conjunction with atezolizumab and taxane chemotherapy. The trial's main objective was not to assess the precise advantages of combining MEK and PD-L1 inhibitors, so even though it demonstrated possible synergy, the question of their additional effect remained unanswered [54, 55].

Immunotherapy and targeted therapies have a great deal of promise to help TNBC patients achieve better results. Even though the findings of previous research have been conflicting, improving patient selection, combination regimen optimization, and addressing present limitations through additional study are necessary to advance these strategies. Dynamic treatment monitoring and the application of novel biomarkers will be necessary to fully realize the potential of these combination therapies.

### Biomarkers of immunotherapy response

Identifying reliable biomarkers to predict the response to ICIs in TNBC is crucial for optimizing treatment strategies. A number of biomarkers have been identified as possible predictors, including TMB, TILs, PD-L1 expression, and deficiencies in mismatch repair (MMR). While these biomarkers hold promise, their use is not without challenges, and ongoing research is needed to refine and combine them for more accurate patient stratification and treatment optimization.

PD-L1 expression has been considered a key biomarker for predicting ICI response, particularly in tumor and immune cells. However, its utility is limited by several factors. Inconsistent results are caused by the considerable variation in PD-L1 testing techniques, such as the distinctions between 22 C3 and SP142 immunohistochemistry (IHC) assays. For instance, a study reported that the SP142 assay was better at identifying 22 C3-positive tumors than the 22 C3 assay was at detecting SP142-positive tumors. The expression rates of PD-L1 for SP142 IC ≥ 1%, 22 C3 CPS ≥ 10, 22 C3 CPS ≥ 1, and 22 C3 IC ≥ 1% were 50.9%, 27.2%, 53.9%, and 41.8%, respectively. The analytical concordance (kappa values) between SP142 IC + and these three different 22 C3 scorings were 73.7% (0.48, weak agreement), 81.5% (0.63), as well as 86.6% (0.73) [48, 56–58]. Furthermore, the predictive value of PD-L1 expression is complicated by its temporal and spatial heterogeneity, particularly in metastatic sites.

TILs have been consistently correlated with better ICI outcomes. TILs have level 1B evidence to predict clinical outcomes in early TNBC, making them a promising biomarker to identify patients who might benefit more from ICIs and have better prognoses with less aggressive cancer treatments [59].

TMB, defined as the number of non-synonymous mutations per megabase, reflects a tumor’s immunogenic potential. A threshold of ≥ 10 mutations/Mb has been associated with improved ICI responses. Based on data from the phase II KEYNOTE-158 study, the anti-PD-1 antibody pembrolizumab was granted approval for treating patients with advanced solid tumors and TMB ≥ 10 mutations per megabase [59, 60]. However, while TMB is a promising biomarker, its predictive value is not absolute, as it does not guarantee a response in all patients, suggesting the need for additional markers to enhance prediction accuracy.

MMR Deficiency and Microsatellite Instability (MSI) are rare in breast cancer but have shown sensitivity to ICIs. The FDA approval of pembrolizumab for MSI-high or MMR-deficient metastatic tumors, based on KEYNOTE-158 data, has highlighted the potential of MMR deficiency as a predictive biomarker for immunotherapy. A study of 316 breast cancer cases found that only four exhibited MMR deficiency, all within the TNBC subtype. Although this suggests that MMR deficiency is infrequent in TNBC, it also highlights the potential benefit of ICIs for these patients [56].

The development of immune gene signatures has shown potential in differentiating “immune-hot” from “immune-cold” tumors. A 12-gene signature, for instance, contains immunosuppressive genes (PDCD1, CD274, CTLA4, FOXP3, IDO1) as well as immune-activating genes (CCL5, CXCL9, CXCL13, CD80, CD21, CD8 A, IGKC). The prevalence of specific immune signatures has rarely been assessed in the function of the TNBC molecular classification [61].

Detection of ctDNA is an emerging non-invasive alternative to tissue biopsy approaches, requiring only a blood sample and allowing collection at different time points with minimal discomfort to the patient. This method enables monitoring of disease progression and response to treatment in TNBC patients, providing physicians with an invaluable tool to tailor treatment to the individual and improve patient outcomes [62].

Advances in understanding cell death mechanisms have identified new potential therapeutic targets for TNBC. One such target is glycogen synthase 1 (GYS1), a key enzyme involved in glycogen synthesis [46, 63, 64]. Targeting GYS1 in TNBC cell lines has been shown to cause F-actin contraction and cell death through a process known as disulfidptosis, which is defined by an excessive buildup of disulfides in cells [64].

While these biomarkers and novel therapeutic targets hold promise, their use is not without challenges. Ongoing research is essential to refine and combine them for more accurate patient stratification and treatment optimization.

Additionally, real-time monitoring of biomarker fluctuations throughout treatment could offer a more precise and adaptive strategy for patient selection. Ultimately, refining these predictive models will be key to optimizing the clinical benefit of ICIs and personalizing therapy for TNBC patients.

## Conclusions

TNBC remains a challenging clinical entity due to its heterogeneity and aggressive nature. However, the advent of ICIs represents a significant breakthrough, particularly for specific patient subgroups, such as those with BRCA mutations. In order to maximize their effectiveness and overcome treatment resistance, ongoing clinical trials are still investigating different combinations of ICIs and targeted therapies. Furthermore, studies on new targets like GYS1 and the TME may yield fresh approaches to enhance treatment outcomes and more precisely tailor treatments for patients with TNBC.

## Data Availability

Not applicable.

## Abbreviations

CHT: Chemotherapy
CPS: Combined Positive Score
CR: Complete response
ER: Estrogen receptor
HER2: Human epidermal growth factor receptor 2
HR: Hormone receptor
ICIs: Immune checkpoint inhibitors
ITT: Intention-to-treat
mTNBC: Metastatic triple-negative breast cancer
MSI: Microsatellite instability
MMR: Mismatch repair
NACT: Neoadjuvant chemotherapy
ORR: Overall response rate
OS: Overall survival
PDL1: Programmed death-ligand 1
pCR: Pathological complete response
PFS: Progression-free survival
PR: Progesterone receptor
RR: Response rate
TILs: Tumor-infiltrating lymphocytes
TMB: Tumor mutational burden
TME: Tumor microenvironment
